# Sexuality and Related Disorders in OCD and Their Symptoms

**DOI:** 10.3390/jcm14196819

**Published:** 2025-09-26

**Authors:** Javier I. de la Iglesia-Larrad, Ramón Kristofer González-Bolaños, Isabel María Peso Navarro, Rubén de Alarcón, Nerea M. Casado-Espada, Ángel L. Montejo

**Affiliations:** 1 Psychiatry Service, Hospital Clínico Universitario de Salamanca, 37007 Salamanca, Spain; rkgonzalez@saludcastillayleon.es (R.K.G.-B.); impeso@saludcastillayleon.es (I.M.P.N.); rubenalarcon@saludcastillayleon.es (R.d.A.); nereacasado@saludcastillayleon.es (N.M.C.-E.); 2 Institute of Biomedical Research of Salamanca (IBSAL), 37007 Salamanca, Spain; 3 Faculty of Medicine, University of Salamanca, 37007 Salamanca, Spain; 4 Faculty of Nursing, University of Salamanca, 37007 Salamanca, Spain

**Keywords:** obsessive–compulsive disorder, sexual dysfunction, sexual obsessions, compulsive sexual behavior, neurobiology, OCD treatment

## Abstract

**Background/Objectives:** Sexuality is a frequently overlooked but clinically significant dimension in patients with obsessive–compulsive disorder (OCD). Beyond comorbid anxiety and depressive symptoms, OCD can substantially affect sexual functioning and include obsessions and compulsions relating to sexual content. This review aims to synthesize current evidence on sexual dysfunction in OCD and the role of sexuality in OCD symptom dimensions, as well as associated neurobiological, cognitive, and clinical outcomes. **Methods:** We conducted a review of the literature including studies published in the last 20 years using the PubMed and Cochrane databases. Our search strategy used the terms “sexual AND (Obsessive-compulsive disorder OR OCD)”, retrieving a total of 582 articles. After a screening and eligibility assessment based on predefined inclusion and exclusion criteria, 200 studies were included. Additional papers were retrieved through citation tracking. **Results:** Sexual dysfunction is highly prevalent in OCD patients, particularly among women, ranging from low desire and arousal to anorgasmia and pain during intercourse. Sexual obsessions affect a large proportion of OCD patients and are associated with an early onset, male sex, greater symptom severity, poorer insight, and suicidality. These obsessions often co-occur with aggressive or religious themes. Neuroimaging studies indicate distinct patterns of brain activation in patients with sexual obsessions. Treatment with SSRIs and CBT is often less effective in this subgroup, suggesting the need for targeted interventions. **Conclusions:** Sexuality-related symptoms in OCD patients constitute a distinct and clinically relevant domain that affects functioning, prognosis, and treatment response. Recognizing and addressing these symptoms is essential for the holistic and effective care of patients with OCD.

## 1. Introduction

OCD has a prevalence of around 1–3% in the general population [1]. Formerly cataloged under the DSM section dedicated to anxiety disorders, OCD constitutes its own diagnostic category in DSM-5, grouped with disorders such as body dysmorphic disorder, trichotillomania, and hoarding disorder, among others. OCD causes major health expenses in relation to mental illness, as well as a significant impairment in functioning, quality of life, and disability [1]. OCD symptoms include obsessions, i.e., repetitive thoughts that tend to be described as ego-dystonic or unwanted, which are intrusive and disturbing to the subject experiencing them [1]. Depression, anxiety, and bipolar disorders are common comorbidities and some of their symptoms can be observed in patients with schizophrenia. The prevalence of depression in OCD ranges from 30 to 80%. Depression caused by OCD can lead to suicidal thoughts, which underlines the importance of the disorder [2].

This review aims to explore sexuality through its different interactions with OCD: both as an impaired function of the patients suffering from the disorder and as a manifestation of the symptomatic presentation of the disease. Sexuality is usually affected by disease, whereby fears of contamination have been related to sexuality problems in OCD patients [3]. The need to avert sexual impulses makes the patients reluctant to take part in sexual interactions. This leads to large rates of sexual dissatisfaction among men and women with OCD [4,5]. Patients express that they experience a decreased libido or interest in their sexual activity, as well as significant feelings of discomfort and avoidant behaviors [5].

According to DSM-5 diagnostic criteria, OCD is characterized by the presence of obsessions and compulsions (or rituals) [1], which are time-consuming and disruptive [6], and may present with a wide range of themes. Obsessions vary in themes and content, but several studies have organized the diverse symptoms within sets of obsession and compulsion clusters [7,8,9,10]. The Yale–Brown Obsessive–Compulsive Scale (YBOCS), the most widely used severity assessment scale in both adults [11] and children [12], distinguishes symptom clusters based on their content and includes sexual obsessions as a distinct and relatively frequent subtype [4]. They include perverse or aggressive sexual thoughts, obsessions about incest or child abuse, and obsessive doubts about the subject’s sexuality, among others [11]. The present review builds on the implication that sexual obsessions— and by extension, sexual compulsions—can be considered as specific symptoms with implications for the course, clinical presentation, and comorbidity of OCD.

The term “compulsive sexual behavior” describes a pattern of excessive sexual behavior, including fantasies and sexual urges that disrupt normal functioning and generate stress [5]. It is not present in all diagnostic manuals [13] and although it is not necessarily related to OCD, its inclusion within a similar category has been proposed [14,15,16,17]. Its nature as a mental disorder has been debated [5,18,19,20,21]. Sexual obsessions have been considered to be “pure obsessional” OCD cases, which is also controverted [22,23].

In recent years, a growing interest in the matter has led to an increased number of publications about the effect of OCD symptoms on sexuality, the description of sexuality-related ideation in OCD, and the effects of treatment on the sexual sphere. Hence, we offer this review with the intention of providing an overall view of the available data regarding this. With our work, we aimed to gather evidence in order to describe the ways in which sexuality is affected by OCD in relation to the following three fundamental aspects: (1) the way in which sexual functioning is affected by the disease, including the presence of sexual disorders but also specific traits that are particular to the sexual lives of OCD patients; (2) how sexuality is involved in the themes of OCD symptoms (obsessions with sexual content and sexual compulsions, as well as the ongoing debate surrounding such compulsions); and (3) how the presence of sexual dysfunctions or sexually themed symptoms affect a varied array of clinical and neurobiological outcomes.

## 2. Materials and Methods

Our design was not that of a full systematic review. However, regarding data collection and screening we incorporated some systematic principles, including predefined inclusion and exclusion criteria and a structured search strategy. We expressed our research question in line with a PICO structure, as follows:

- P (Population/Problem): Patients diagnosed with obsessive–compulsive disorder (OCD), including both adults and adolescents, particularly those exhibiting sexual symptom dimensions. We aimed to include the widest possible variety of OCD presentations to examine how symptomatology related to sexual functioning and impairment. No age or demographic restrictions were applied, in order to capture evidence on sexual symptoms and impairment in OCD across diverse populations and to account for predictable variability.
- I (Intervention/Exposure): Presence of sexual dysfunctions, sexual obsessions, sexual compulsions, and compulsive sexual behavior within the OCD spectrum.
- C (Comparison): General population and individuals diagnosed with other psychiatric conditions (e.g., major depressive disorder, generalized anxiety disorder, panic disorder, and schizophrenia).
- O (Outcome): Altered sexual functioning (e.g., libido, arousal, orgasm, and satisfaction), psychological distress and functional impairment, treatment response (e.g., CBT, SSRIs, EMDR, and DBS), and neurobiological and cognitive correlates.

**Figure 1:**
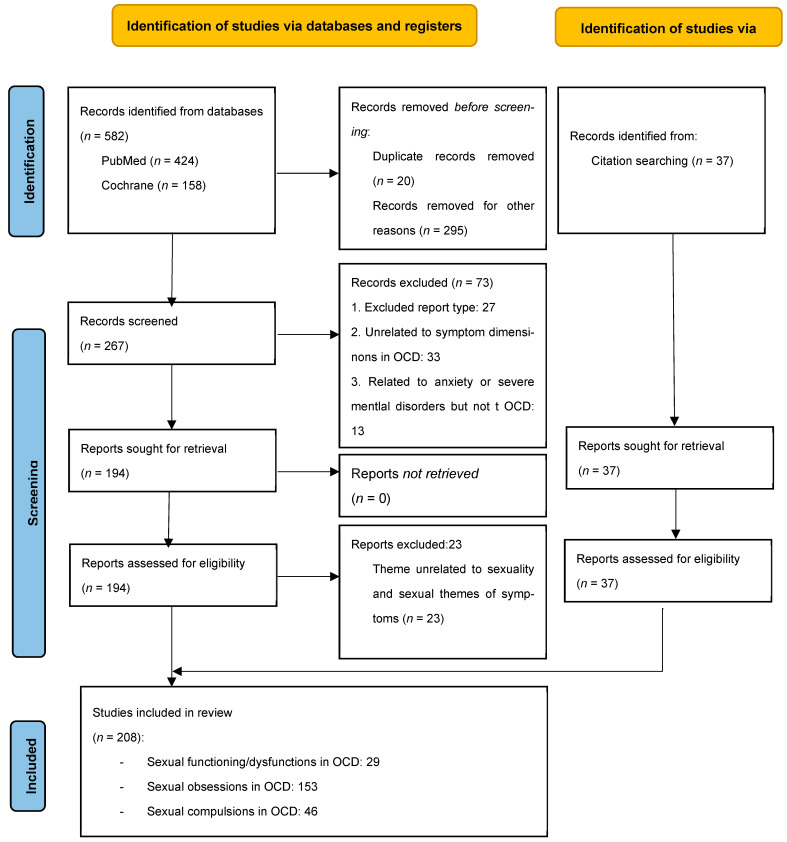
Flow diagram showing the identification, screening and inclusion process for the articles selected for the review.

Our search was initiated on 31 January 2024. We addressed our population and their exposure to the core symptoms of OCD through terms including OCD, obsessions, and compulsions, while their sexual content and outcomes related to sexuality were addressed with the term “sexual”. We used the following search strategy: “sexual AND (“Obsessive–compulsive disorder” OR OCD)”, limiting the search to studies published in the last 20 years. The search yielded 424 results in PubMed and 158 entries in the Cochrane database. After removing duplicates, we reviewed the titles of these entries in search of articles that contain data related to the themes of the review, including articles covering a wide range of mental disorders, particularly anxiety disorders (given the inclusion of OCD within that category prior to DSM-5). The inclusion criteria can be seen below. We included 267 papers for abstract screening. We excluded the following: 27 papers that did not fit into any of the study types considered for inclusion; 33 studies about OCD with abstracts (though not titles) that specified objectives of study that were completely unrelated to sexuality or OCD symptom dimensions; and 13 studies about severe mental disorders whose abstracts did not mention OCD when listing the disorders taken into consideration. A total of 194 papers were assessed, 23 of which were excluded since they did not include any information regarding sexuality itself or sexual obsessions or compulsions in OCD, even if they were about OCD or sexuality in mental disorders. Through citation searching within these articles, we identified 37 additional papers. We included 161 papers from the database search and 37 additional papers identified through citation searching, resulting in a total of 208 publications being included in our review (see Figure 1).

Inclusion criteria

- Titles or abstracts referring to sexual functioning in OCD. During title or abstract screening, every title referring to sexual functioning or dysfunction in severe mental disorders or anxiety disorder was included for further assessment.
- Titles/abstracts about symptom dimensions and OCD, including obsessions and compulsions.
- Titles or abstracts including symptoms or phenomena in the sphere of OCD, such as “subthreshold obsessional symptoms” or “mental contamination/pollution”.
- Titles/abstracts concerning OCD, not limited to a concrete aspect of the illness or the patient’s lives other than sexuality or sexually themed symptoms.
- The studies assessed had to be categorized as a meta-analysis; a systematic or narrative review; or an experimental (randomized clinical trials), observational (case–control, cohort studies), or descriptive study (excluding case reports/series, except in animals).

Exclusion criteria

- Case reports, series of cases, studies in animals, correspondences, editorials, and any other types of study not listed within the inclusion criteria.
- Studies concerned with sexuality but not mental disorders, or with a specific aspect of mental illness or OCD unrelated to sexuality.
- Articles specifically about sexual dysfunctions caused by the treatment of OCD, i.e., sexual dysfunction that is secondary to treatment with clomipramine or serotonin reuptake inhibitors (SRIs) for OCD.
- Studies concerned with sexuality in other specified mental disorders other than OCD.
- Papers concerning OCD but focusing on other symptoms other than those that are sexually themed (for example, only measuring the general severity of the symptoms without addressing sexual obsessions or taboo/unacceptable thoughts separately).
- Statistical signification is either not clearly stated and justified via statistical analysis or inference in reports establishing associations between variables, or the methods to identify the association are not specified.

## 3. Results

### 3.1. Sexual Functioning in Obsessive-Compulsive Patients

#### 3.1.1. Sexual Development

Earlier menarche ages were reported in OCD patients [24]. Nocturnal ejaculation was more common in OCD males than in controls [24]. OCD patients have been reported to have their first sexual intercourse later in their lives, compared with controls [4,25] and patients with anxiety disorders [25] or PTSD [25], but at a similar age to those with GAD or depressive disorders [26]. Belonging to a sexual minority has been found to be correlated with greater emotional regulation difficulties among patients with OCD [27]. In another study, young OCD patients and controls seem to have similar feelings of guilt over masturbation, the frequency and age of homosexual experiences, and kissing and petting experiences [4]. No difference was found between the age of first sexual impulses [4] and the age at which the study participants learnt about sex [26].

For OCD patients, it has been found that sex was not discussed openly or frequently in their childhood homes [4,26]. However, it was also not discussed in the homes of patients with depression or panic disorder; therefore, this trait may not distinguish OCD patients [26].

In a Brazilian study, patients with OCD were found to present with a history of sexual abuse during childhood at a higher frequency than patients with general anxiety disorders [28].

#### 3.1.2. Social and Marital Difficulties

Some earlier studies documented a tendency towards heterosocial isolation in OCD teenagers, with a low frequency of dating and the absence of steady partners, possibly contributing to later sexual difficulties [4,29] In other studies, OCD patients’ degree of comfort on a date was not found to be different from that of depressive and anxious patients [26]. In some studies, most OCD patients reported relationship problems that lead to poor-quality sexual interactions [4,30]. However, OCD patients were not found to be more dissatisfied with their marriage relationships than other diagnostic groups, including patients with depression or anxiety [26]. Indeed, depressed patients had significantly lower levels of marital adjustment than either obsessive–compulsive or panic-disordered patients [26]. Patients who tend not to speak about their symptoms with their partners tend to disclose less personal information, usually out of embarrassment and shame and perhaps because of fear that their symptoms will be held against them [31].

#### 3.1.3. Sexual Excitation and Inhibition Within the Dual-Control Model

Remarkably, and in contrast to the typically emphasized pattern of sexual avoidance in OCD, patients showed significantly higher levels of sexual excitation than controls [32,33,34]. It has been proposed that the suppression strategies used by OCD patients are potentially met with the rebound of sexual fantasies/thoughts, thus lowering the threshold of sexual excitation. Additionally, alexithymic OCD patients may misinterpret arousal-related bodysignals, confusing anxiety and excitation; however, the effect of interpersonal isolation, social avoidance, and depressive symptoms has been suggested to play a role, since depressive patients also demonstrate increased sexual excitation in some studies, perhaps as a strategy to cope with negative moods or as a path to a transient reduction in anxiety and obsessions [32]. In a study comprising a sample of 245 HIV-positive men having sex with men, having OCD was associated with a greater frequency of methamphetamine use, more depressive symptoms, and less sexual activity in the previous months, as well as with more risky sexual behaviors when using methamphetamine [35].

However, OCD patients with more intense symptoms had lower levels of sexual excitation [32,34,36]. Compulsive washing has also been related to experiences of lower excitation, perhaps because the symptomatology focuses on interpersonal contact and the risk of contagion, with sexually transmitted diseases creating an intense experience of disgust and also a stronger focus on bodily signals, subsequently leading to an avoidance coping mechanism that protects the patient from external and internal triggers of obsessions [34]. A tendency for higher levels of sexual excitation has been reported among women with OCD compared to men with OCD [33,37].

Lower sexual excitation levels have been described in women with OCD who had a greater discomfort with closeness or attachment avoidance [33]. However, women who avoided attachment or were uncomfortable with closeness but also had a higher confidence seemed to have higher levels of excitation [37].

Patients with OCD also have greater sexual inhibition than the controls [38], as well as a more stereotypical view of sexuality, which is more immature with the increasing severity of symptoms [38]. OCD patients showed significantly higher levels of inhibition due to the threat of performance failure, as well as inhibition due to the threat of performance consequences, than the controls [32,33,34]. Sexual inhibition is especially relevant in those patients with higher levels of disgust sensitivity, particularly in those patients with contamination obsessions or washing compulsions (which, in turn, are strongly associated with a higher level of disgust sensitivity) [34,36]. There is a correlation between disgust propensity towards pathogens and sex, which is also related to a restriction in sexual strategies in patients with OCD; this has been hypothesized to restrict sexual contact even when their disgust is not related exclusively to diseases that are not transmitted sexually or with sex itself [39]. Patients with more severe symptoms of checking compulsions also reported a more elevated inhibition, which is specifically due to the threat of performance consequences (e.g., undesired pregnancy and/or sexually transmitted diseases) [34]. Patients with higher levels of perfectionism also showed a greater sexual inhibition due to the threat of performance failure [33,34].

Women reported lower levels of sexual inhibition due to the threat of performance consequences than men, especially those with attachment anxiety [37], less confidence [33] and contamination obsessions, which seemed to be more frequent in women with OCD [37]. OCD patients are very sensitive to mild relationship or sexual implications, even those within the range of social acceptability, tending to react with feelings of guilt [38] (see Table 1).

#### 3.1.4. Sexual Dysfunction and Dissatisfaction

SD is considered an especially common finding in patients with OCD [3,4,24,30,40,41,42,43]. Global prevalence rates for sexual dysfunctions among OCD patients have been reported to range between 39 and 77.5% [3,4,24,30,40,41,44]. When divided by genre or in studies comprising only women with OCD, prevalences range between 25% and 81% [3,4,24,30,40,41,42,43,44]. The available literature agrees that sexual dysfunctions are significantly more common in OCD patients than in healthy subjects [3,4,30,42,44], with the prevalence of SDs that are observed in the general population being between 10 and 40% [34]. (See Table 2, Table 3 and Table 4).

In general, comparisons regarding mental disorders and OCD tend to differ [44]; however, they are recognized as an impactful issue, perhaps not more common than in other mental disorders [26,44], and have a significant effect on sexual satisfaction in these patients [4,26,40,43]. Significant correlations between the presence of SDs, quality of life, and satisfaction scales indicate that sexual disorders have a negative effect on happiness; life satisfaction; and the sense of strength, meaning, and control in patients with OCD [30].

In studies on female patients, high prevalences of SDs were reported [3,43,45]; according to some studies, these are found to be more common in women with OCD than in men [24,30,40], although not all of the evidence supports this comparison [41,44]. Global SD prevalences in OCD range from 24 [24] to 81% [40] for women and from 25 [40] to 64% [41] for men.

Some authors interpret that sexual conflict or dysfunction may be associated with psychopathology, in general, rather than with OCD [26]. However, SDs have been reported to be significantly more common in OCD patients than in those with other mental disorders. Higher prevalences of SDs were found in patients with OCD than in those with panic disorder, especially among women, [3] but were found to be lower than in patients with GAD [44]. SDs are more common among OCD patients with depressive symptoms [40], although patients with major depressive disorder had more SDs than OCD patients [44]. Some authors report higher SD rates for OCD than for schizophrenia [4,42]. Patients with schizophrenia might be less capable of detecting, reporting, and associating their sexual dysfunctions with their mental symptoms, possibly due to cognitive disabilities associated with schizophrenia [4].

##### Low Frequency

Infrequency seems to be a common complaint among OCD patients [4,5,41,46]. In one study, a quarter of OCD patients reported having had no sexual intercourse for many months prior to the interview [4]. Occasionally, this has been attributed to a feeling of aversion to sex in OCD patients [5,41].

Female OCD patients complain more commonly of the infrequency of sexual intercourse in studies comparing samples of different anxiety disorders with mixed genres [24]. However, it has been reported to be more evident (severe) in male patients with the same complaint [24]. Studies from Aksaray et al. (2001) [45] and Aksoy et al. (2012) [24] suggested that OCD patients, in general, had more infrequency problems than patients with generalized anxiety disorder [45] and panic disorder [24], respectively [32].

We found few studies directly measuring the frequency of sexual intercourse among OCD patients [24,32,45,46] or their complaints about this issue; comparisons with other disorders rely on studies with small samples. However, the results suggest that OCD patients could have sexual relationships less frequently than patients with anxiety disorders such as PD or GAD.

Although some studies have found no correlation between the severity of OCD symptoms and the occurrence and intensity of sexual dysfunctions in general [44]—particularly in anorgasmia [45,47]—other earlier studies found that OCD patients felt that there was a relationship between the severity of OCD and their sexual symptoms [4]. In the same studies, it was noted that patients with schizophrenia did not seem to find such a strong influence of their mental illness on their sexual performance [4], presumably—according to the authors—due to their greater cognitive impairment, making it impossible for them to associate their symptoms with sexual difficulties [4].

Fears of contamination from sexual activities appear to correlate with more severe cases of OCD [31]. Contamination obsessions and washing compulsions in patients with greater disgust sensitivity were correlated with higher levels of sexual inhibition due to fear of performing badly during intercourse and due to a fear of its consequences [36]. In patients centering their disgust or apprehension on aspects related to the sexual sphere, researchers were more likely to find a propensity towards feeling mental contamination (feeling contaminated without direct interaction with the source of disgust but merely seeing or thinking of it) [39]. In those patients with obsessions centered around the patient’s romantic partner, sexual satisfaction was found to be decreased; however, this relationship was mediated by relationship satisfaction [48].

The presence of OCD, its duration, the number of comorbid mental disorders, the general severity of OCD, and the number of anankastic personality traits have all been found to correlate with the ASEX score [30]; some other studies have found sexual excitation to decrease with the severity of OCD symptoms, perfectionist/anankastic beliefs, or personality traits [32]. OCD patients have a reduced sexual experience and dissatisfaction, which is reported to be related to the disease process itself [40]. Comorbid depressive symptoms (in addition to prompting erection difficulties) also worsen sexual satisfaction [40]. The severity of obsessions was associated with lower levels of intimacy between the patients and their partners, independent of depressive symptoms [31]. However, compulsions of washing and neutralizing were associated with increased levels of intimacy and satisfaction with their relationships [31].

##### Aversion, Avoidance, and Impaired Sexual Desire

Infrequency has been attributed to sexual avoidance in OCD patients [5,41]; this has been described as being common for OCD patients [24], especially among females [24,43,45]. This phenomenon seems to be more common in OCD patients than in those with other anxiety disorders but could be less frequent than in patients with panic disorder [24]. This has been attributed to the absence of a partner [41], although it could be related to problems with sexual arousal [24,43] and particularly with contamination obsessions [32].

Sexual infrequency and avoidance could be related to feelings of aversion towards sex in OCD patients [46], which, in some studies, appeared to be less frequent in male patients [3]. However, this trait might not be exclusive to OCD patients and has been reported as being more frequent in other anxiety disorders [3]. In OCD, sexual aversion seems to be related to contamination obsessions and washing [36], especially in patients with increased levels of disgust sensitivity [36] and mental contamination feelings (e.g., fear of contamination by merely observing or thinking about something unclean or immoral) [3,39]. A proneness to disgust that is focalized in infectious diseases (which are not necessarily transmitted through genital contact) may be more frequently associated with sexual aversion [39]. This seems to cause more sexual inhibition due to the threat of performance failure and a fear of its consequences [36]. Homosexual fantasies or thoughts may be related to feelings of guilt [4]. Additionally, given their fear of interpersonal contact, more intense feelings of disgust, and a strong focus on bodily signals, patients with more severe symptoms of compulsive washing experienced lower levels of excitation [34]. This might be a coping mechanism against sexual fantasies, internal triggers for fears of contamination, and washing compulsions [34].

Decreased levels of sexual desire seem common among OCD patients, having been described as being more severe and common among women with OCD than among male patients [3,30,43]. However, the results diverge [42]. Although contamination obsessions seem to be related to greater levels of sexual inhibition and a fear of the consequences of sexual interactions, washing compulsions have been found to be related with increased levels of sexual desire [31]. Decreased levels of sexual desire appear to be more common in OCD patients than in those with GAD [45].

##### Arousal

Patients with OCD showed lower levels of sexual excitement and arousal than the controls [3,24], especially for women [24,30,43], who were described as being “more nonsensual” in some studies [24,45]. The fear of contamination has also been related to being “nonsensual”, while washing compulsions have also been related to sexual inhibition in OCD patients [24] and to lower levels of excitation [34]. Disgust sensitivity has also been found to be related to a decreased sexual arousal capacity, leading to the hypothesis that disgust sensitivity produces an increased focus on the body that distracts from arousal-inducing feelings [36]. This lack of arousal might be independent from the severity of OCD symptoms [36]; it is more common in OCD patients than in those with GAD and PD [24].

##### Erectile Dysfunction and Premature Ejaculation

The ability to achieve an erection is one of the most common sexual dysfunctions in men with OCD [4,24,28,30]. This phenomenon seems to be independent from sexual desire [49]. The presence of concomitant depressive symptoms worsens erectile dysfunction [40]. Some studies have found similar prevalences of ED in other psychiatric conditions [49], while others have found that among patients with OCD, ED was more severe than in patients with GAD [28]. As discussed previously, although premature ejaculation has been reported more commonly among SAD patients [28], it is also common among OCD (and PD) patients in comparison to the general population [4,24].

##### Orgasm Dysfunction, Lack of Pleasure, and Pain During Intercourse

Patients with OCD and anxiety disorders most frequently reported dysfunction in the orgasm phase, [42] although these dysfunctions seem to be especially frequent among patients with OCD [4,42], with higher rates of orgasm dysfunction than in patients with GAD [28,44], PD [24], or social anxiety disorder (related to premature ejaculation in male patients) [28]. Rates for orgasmic dysfunction or anorgasmia in OCD patients have been reported to be comparable or even higher than those in depressive patients [44]. Its severity appears to be similar in OCD patients and in those with GAD or MDD [44] and it does not appear to depend on the severity of OCD symptoms [46]. It tends to be one of the most common kinds of dysfunction in many patients, both for men [44] and women [24,30,43], or the most common type [42,44], with complete anorgasmia being more common in women [45]. This increased prevalence of orgasm impairment might be related to the necessity of OCD patients to keep their thoughts under control [28]. According to various studies, women very often reported a lack of pleasure from orgasms, which sometimes constituted the most frequent complaint [30,43]. Diminished vaginal lubrication also seems common among women with OCD [30]. Female OCD patients had higher rates of vaginismus and pain during intercourse [4] than controls and patients with PD and GAD [24]. Male patients have been reported to show less genital pain than women with OCD [3,30].

##### Dissatisfaction

Sexual frustration and dissatisfaction are common in people with OCD [4], as is also the case for patients with depressive and anxiety disorders such as PD [24,42], for which similar prevalences of sexual dissatisfaction tend to be reported [26]; hence, it may be related to mental illness, in general, rather than to mechanisms specific to OCD, as has been suggested previously [26]. In OCD patients, sexual frustration and dissatisfaction appear to be independent of the presence of mood or anxiety symptoms [24]; however, comorbid depression has been found to worsen dissatisfaction [40] and is not strictly related to the presence of specific sexual dysfunctions, since its prevalence tends to be higher than the global rates of SD [44]. Sexual dissatisfaction has been associated with a feeling of guilt [4]. Some studies found it to be more frequent in women [24], specifically comparing women with OCD to those with panic disorder [3].

### 3.2. Sexual Obsessions

#### 3.2.1. Definition of Sexual Obsessions

A study conducted on the general population in relation to intrusions and their relation to obsessions allows for a differentiation between two kinds of thoughts—morally based or non-moral obsessions [50]. A continuum between such thoughts and OCD obsessions was suggested by the characteristics and associations of the intrusions [50]. Sexual obsessions are usually classified under the broader category of unacceptable thoughts, but there is evidence that hints that it might be more beneficial to separately address sexual thoughts through specific scales [51]. Sexual obsessions (SO) include fears of committing a sexual aggression [51,52], pedophilic sexual obsessions [53], and pathological doubts about one’s sexual orientation [54]; some kinds of transformation obsessions can include the fear of a genital or gender-related sexual identity transformation [55,56], while romantic obsessions involve excessive ruminations over the feelings of those involved in a relationship, including doubts about physical attraction [48].

#### 3.2.2. Epidemiology

According to the reviewed literature, up to two-thirds of OCD patients suffer from sexual obsessions [52,57,58,59,60,61] (See Table 5). Sexual obsessions are more common in males than in females, as has generally been shown by the evidence gathered in relation to this issue, which has been replicated in samples from all age groups and varied nationalities [10,62,63,64,65,66,67,68,69,70,71] with a minority of discrepancies [23,60]. As a symptom dimension for OCD, it is not among the most common obsessions in undiagnosed children [72]. Men tend to demonstrate an earlier age of onset [10,65] and a chronic course of OCD [65]. Symptom presentations for OCD patients seem to be hereditary to some degree; therefore, sexual obsessions may be similar [73,74]. However, in a comparative study, sporadic OCD patients were reported to have more sexual obsessions than hereditary OCD patients [75]. One study researched specific intronic variants of the serotonin transporter gene but did not obtain significant results [76].

#### 3.2.3. Clinical Course

Although not every study found a correlation [114], sexual obsessions were associated with an earlier onset of OCD symptoms (especially, but not exclusively, in male patients) [9,64,87,102,111,115,116,117]. Early-onset OCD was also correlated with being male [115], a family history of OCD [87,102,115], more aggressive obsessions [102], pathological doubts [102], tics [115], and repeating rituals [102]. Patients with early-onset OCD tend to have a chronic course of the disorder and a greater social impairment [65], as well as a lower insight into sexual/religious obsessions [116,118]. Symmetry/exactness obsessions and washing/cleaning compulsions were also correlated with early-onset OCD in some studies [111], whereas later-onset OCD appeared to be correlate obsessions [117]. Sexual obsessions were associated with an earlier interference with functioning [10], a greater severity of general OCD symptoms [95], and a poorer social function [99].

Alexitimia seems to be frequent in juvenile OCD patients with sexual obsessions [119]. Among young patients with OCD, it was those patients over 9 years old [100] that showed an increased occurrence of sexual obsessions, especially around the age of 15 [23], whereby magical thinking [23,100]; somatic obsessions [100]; checking and counting [100]; staring and blinking rituals [23]; and comorbid depression [23,100] were prevalent. However, an Iranian sample of more than 200 patients showed a different distribution of the symptom dimensions among clusters, excluding the aggression/religion/sexual cluster and suggesting an influence of culture on symptom manifestation in OCD patients [79]. The association between age of onset and sexual obsessions seemed to suggest that such content might be linked with the usual worries that are typical of the corresponding age group; however, there is a lack of evidence to support this claim [120] as obsessional themes do not seem to vary throughout age groups, and since unacceptable, religious, and hoarding symptoms have shown a significantly high temporal stability through adulthood [81,121]. Some authors have proposed that childhood-onset OCD might be a different [121] neurodevelopmental variant of OCD [38].

Sexual obsessions are among the kind of obsessions that cause most shame among the affected patients [110,122], significantly more so than in relation to contamination and symmetry obsessions [110]. They cause great distress and are associated with affective symptoms [110]. However, in one study, they seemed to be the second-least-severe symptom dimension [123]. Taboo obsessions or unacceptable thoughts, including sexual obsessions, have been identified as independent risk factors for suicidality in OCD patients [68,83,108,124,125,126]. Some studies found the contribution of taboo or sexual obsessions to be milder in comparison with other factors, although still significant [83], while others suggest a high risk of suicide to be associated with these specific contents [90,125,127]. Although rates of suicidality were lower in OCD patients, in general, than in patients with other mental disorders such as schizophrenia [124], rates of suicidal ideation in OCD patients with sexual obsessions of 33% have been reported in different studies [2,124].

In the same studies, other factors accounting for suicidality in OCD were the general severity of OCD [83,108], lifetime major depression [83,108], severity of depressive symptoms [126], lifetime substance use disorders [83], a poor subjective quality of life according to the SF 36 QoL scale [126], a history of previous suicide attempts in the family [108], a history of personal childhood trauma or sexual abuse [126], and impulse–control disorders [108].

#### 3.2.4. Psychological Patterns, Beliefs, and Upbringing

Mental stiffness was a frequent trait of OCD patients with sexual obsessions [10]. The cognitive theory of obsessions suggests that obsessions are caused by appraisals made by the patients about intrusions that are otherwise widespread throughout the healthy population [128]. In the case of unacceptable thoughts, appraisals or beliefs [103,128]. The same appraisals seem to mediate sexual intrusions in the general population with a background of sexual trauma [129]. Intrusions of sexual content tend to be less negatively appraised than violent intrusions [130] and more so than blasphemous content [128]. Sexual/religious obsessions were distinctly correlated with two early maladaptive schemas—vulnerability to harm or illness and enmeshment/undeveloped self—independently of clinical variables such as the severity of OCD and duration of illness [95]. Inflated responsibility beliefs have been associated with romantic OCD [7] and sexual obsessions in general [7]. In romantic OCD, less perfectionism and an overestimation of threat have been measured in comparison with other OCD patients [131].

In another study, 146 OCD outpatients were compared to 80 healthy controls and were evaluated using the Sociodemographic Data Form, SCID-I, SCID non-patient version, the Yale–Brown Obsessive–Compulsive Scale (Y-BOCS), and the Barratt Impulsiveness Scale (BIS-11), showing a correlation between impulsivity and sexual obsessions [132]. Anger, aggressiveness, and impulsivity were associated with sexual obsessions in one study from 2016 [133]. Taboo thoughts were linked to greater levels of hostility [134].

A study on the influence of parental traits failed to associate sexual obsessions with any specific trait (only hoarding was linked with a low parental warmth) [135]. All the unacceptable thought obsessions were associated with a history of childhood trauma and, at the same time, with ritualistic compulsions [82]. Clinicians may suspect child abuse as a background for sexual obsessions [23]. Some published cases can be found relating to children with obsessions of sexual abuse [23,136]. A correlation between a background of abuse and OCD has been described [137,138]. The evidence in relation to that is still conflicting; thus, reviews exploring the matter recommend to suspect child abuse when sexual obsessions are present in pediatric patients, without excessively insisting the exploration of such an area, indicating that this could be harmful for the patient [23]. It has been pointed out that obsessions relating to unacceptable thoughts could cause mental contamination following sexual trauma, particularly if the patient perceives that the experience makes them sinful or immoral [139]. In pediatric samples, sexual and aggressive obsessions have been associated with a fear of separation, with these results being replicated in different studies [23,62,67,68,70,71].

#### 3.2.5. Neurobiological Correlations

Some studies have found a particularly significant correlation between sexual/aggressive/religious symptoms and brain activation, with a greater severity of such obsessions predicting a more intense activation of the left anterior insular cortex, ventrolateral prefrontal cortex, left dorsolateral prefrontal cortex, right amygdala, and dorsal anterior cingulate cortex in functional resonance neuroimaging [140]. Sexual symptoms have also been related to more pronounced alterations in the function and structure of the amygdala [141].

In a study consisting of 69 patients with OCD undergoing examination through magnetic resonance imaging (MRI), a greater component of activation in the right amygdala was found in patients with aggression, checking, sexual, and religious obsessive symptoms. Activation in the medial region of the amygdala and prefrontal left cortex was associated in a more specific way with religious and sexual obsessions. These symptoms were negatively associated with the activation of the visual cortex and right fusiform gyrus [142]. A recent study states that in contrast with patients with other symptom dimensions, patients with sexual/religious obsessions were shown to have a greater connectivity between the ventral caudate and the middle and Antero basal insular cortex [47]. Another study found that sexual/religious obsessions predicted the magnitude of amygdala–paralimbic activation [140]. Sexual obsessions could be related to the Val66Val genotype of the BDNF factor [96] and G protein-mediated processes [48,77].

#### 3.2.6. Association with Other Obsessions

Sexual obsessions appear to be mostly associated with religious [9,10,60,65,88,143] or aggressive [60,88,143] obsessions, and usually with both [9,60,66,87,105,107,113,121,144,145,146]. Obsessions of such content seem to share the demographics of sexual obsessions; whereby religious and sexually themed obsessions were both exclusive to male and early-onset OCD patients in one study [143]. Some studies also relate them to somatic obsessions [9,60,66,106,144,145,147] and themes of symmetry [10]. These clusters of symptoms retain their validity for children and adolescent patients [106,148] and across samples of patients with comorbidities such as schizophrenia [88].

In a study centered around the analysis of semantic linkage between self-reported obsessions, sexual harm themes appeared to be associated with themes of contamination, physical harm, and somatic themes [9,66,106,149]. All clusters resulting from the analysis of all kinds of obsessions seemed to converge around the idea of harm [149] (see Table 6).

#### 3.2.7. Association with Other Pathologies

Sexual obsessions are associated with mood disorders [9,109,119,134,161]. According to Hong et al. (2004), sexual obsessions are more common among men with recurrent major depressive disorder [161] and low mood usually appears to be corelated with depressive disorders [9]. A study from 2012 suggested a link between early-onset OCD, sexual obsessions, and alexithymia [119]. Positive correlations were found between patients with OCD, bipolar disorder [162], and cyclothymia [150]. However, in another study, patients with a comorbid bipolar disorder showed significantly lower rates of male gender, sexual and hoarding obsessions, repeating compulsions, and lifetime comorbid substance use disorders and an increased risk of suicide [98].

Anxiety disorders are also frequently associated with sexual OCD [9,88,161,163], such as post-traumatic stress disorder [69], panic disorder/agoraphobia [9], social phobia [109], separation anxiety disorder [109], and generalized anxiety disorder [9]. Other pathologies found to be associated with sexual OCD include any somatoform disorder [9,108,109]. Sexual obsessions were more frequent [164] and severe [65] in OCD patients who had initially received a diagnosis of separation anxiety, who also had a higher risk of experiencing a post-traumatic stress disorder [164]. At the same time, the presence of unacceptable thoughts in OCD patients predicts daily PTSD symptoms [139].

Tic disorders are also frequently correlated with sexual obsessions [69,109,134], although other studies point at a negative association in younger patients [107]. Patients with tics had more severe sexual obsessions [165], as well as experiencing alcohol [9] and non-alcoholic substance abuse [9,108,134]. In Japan, a study carried out in a sample of 41 patients showed a significant correlation between sensory phenomena measured using the Sao Paulo Sensory Phenomena Scale, tics, and sexual aggression obsessions [166]. The obsessions of OCD patients with comorbid Tourette Syndrome tend to have a sexual content [165,167] more often than in patients without comorbidity or with comorbid autism spectrum disorders [97], and more severe OCD [165,167]. Patients with comorbid OCD, sexual obsessions, and Tourette Syndrome seem to present with hoarding symptoms [165] and ordering compulsions more often [97].

Patients with comorbid body dysmorphic disorders seem to have sexual obsessions more frequently [9,86,109] and to be, in general, younger, more frequently single, and unemployed, as well as experiencing higher rates of suicidal behaviors. One study also explored the association between patients with comorbidity and mood, anxiety, and eating disorders, as well as hypochondriasis, skin picking, Tourette syndrome, an earlier onset of obsessive–compulsive symptoms, a greater severity of OCD, depression, anxiety symptoms, and poorer insight [86].

In one study, young OCD patients with ADHD appeared to have fewer sexual obsessions [89]. However, in an adult OCD sample, a previous ADHD diagnosis was found to be associated with higher rates of sexual and religious obsessions, as well as with comorbid bipolar disorder, social anxiety disorder, Tourette’s disease, and with an episodic course of OCD [82].

In patients with comorbid schizophrenia, an association has been described between presenting sexual/religious/aggressive obsessions and the intensity of positive symptoms [88]. According to one study, sexual obsessions are among the most common manifestations of clozapine-induced OCD [91].

#### 3.2.8. Subtypes of Sexual OCD

Transformation obsessions imply the fear of becoming another person, an animal, or seeing one’s body mutated in some form; this includes fears of having one’s sexual organs, characteristics, or identity mutated. One study about comorbidities and associations of these thoughts concluded that transformation obsessions fell into the same symptom cluster as other sexual obsessions or “forbidden thoughts” [54]. The patients tend to be younger, and their diagnosis seems to be delayed, presenting with a greater severity of sexual/religious dimension obsessions and increased suicidality [55].

Sexual orientation obsessions seem to be present in 4–10% of the patients in studies with large samples [114,168], with around 8% labeling these as past symptoms [114]. These obsessions were more common in men than in women [114]. Homosexual obsessions are commonly mistaken by clinicians with concerns about one’s own sexuality [136,169,170], although patients presenting with these in the course of the illness tend to have a moderate severity of OCD symptoms in general [22,114]. Studies on large samples indicate their association with great distress, often leading to suicide [22,171]. The relationship between intrusive thoughts of this kind and the patient’s wellbeing appears to be mediated by psychological inflexibility [172] and disgust sensitivity [54] rather than by dysfunctional beliefs, which leads to the interpretation of homophobic feelings as a disgust response to the obsessions in the treatment of the disorder [54].

Romantic OCD (ROCD) involves intrusive thoughts about the subject’s feelings or the partners’ reciprocal love, inducing the constant doubting and checking of such emotions, which can undermine the relationship and lead to significant romantic dissatisfaction, subsequently leading to sexual dissatisfaction independently of concomitant mood disorders, general worry, or symptom severity [48]. A “contagious” effect of unmediated exposure to partners’ ROCD has been described [173].

#### 3.2.9. Impact of Sexual OCD

There is some evidence that sexual obsessions are among the factors that contribute the most to the identification of OCD [101]. However, according to the available literature, sexual obsessions (and taboo thoughts in general) are often misidentified by non-specialized clinicians [136,169,170,174]; one study from 2017 showed that mental health professionals tend to be biased toward stigmatizing certain intrusive thoughts, including sexually themed ones [175]. Pedophilic obsessions are also usually misunderstood by the general population [53]. Some studies underline the need of not misunderstanding sexual obsessions with doubts on sexual identity or a restrained inappropriate sexual desire (e.g., pedophilia) [23]. It is important to normalize the symptoms in the context of OCD to reassure the patient in relation to dealing with their fears of acting according to their obsessions [23]. This might also influence adherence to exposition and response prevention therapy. When sexual thoughts correspond to ego-syntonic tendencies, the subject would normally try to avoid recognizing them, usually showing no distress as a result of their thoughts, which is in contrast to obsessional symptoms [23].

Sexual obsessions are more stigmatized than religious obsessions or schizophrenia, according to one study on the general population [176]. It has been pointed out that ERP directed towards sexual orientation obsessions tends to increase homophobic attitudes; recommendations have been given to improve that approach in a socially just manner [177].

#### 3.2.10. Treatment of Sexual OCD

Current guideline-based care for OCD and BDD follows a stepped-care framework [52,178]. First-line psychological treatment is cognitive-behavioural therapy with exposure and response prevention (CBT-ERP), with low-intensity formats for mild impairment and more intensive individual CBT-ERP for moderate to severe presentations. Selective serotonin reuptake inhibitors (SSRIs) are an evidence-based alternative to CBT-ERP for moderate impairment and are combined with CBT-ERP in severe cases; clomipramine is considered after inadequate SSRI trials [1,178]. In children and adolescents, CBT-ERP adapted to developmental level and involving families/carers is first-line, with cautious SSRI use and close monitoring when indicated. Poor responders should be reviewed by specialist multidisciplinary teams, with options including additional/intensive CBT-ERP, pharmacological optimization or augmentation, and—in highly refractory adult OCD—referral to tertiary services. Because sexual symptomatology can be associated with poorer response to standard interventions, systematic assessment of sexual symptoms may help personalize treatment choice and intensity within this stepped-care model.

The efficacy of pharmacotherapeutic treatment and behavioral therapy has often been researched and established, estimated as being around 85% for OCD patients, in general [52]. Sexual obsessions have been identified as a factor predicting treatment refractoriness [179]. There is conflicting evidence about medical treatment effectiveness, whereby a better response to citalopram [104] or other agents was predicted in some studies according to sexual obsessive contents [141,180]. These results have been replicated in pediatric patients [85], but many authors indicate a poorer response to SRIS in patients with sexual obsessions [93,150,181,182]. According to the results of a meta-analysis carried out in 2015, the presence of sexual/religious obsessions and compulsions was associated with a significantly better response to deep brain stimulation (DBS) [183]. Sexual OCD showed a better response to surgery than OCD patients with other obsessive themes [184].

Concerning behavioral therapy, sexual obsessions have a worse response [155] and take longer to improve [52,78], although the results are controverted [185]. Due to the dominance of obsessions in these patients, exposure response prevention (ERP) therapy may be less effective than in other patients with OCD [52,186,187]. Some authors recommend the introduction of methods based on attentiveness and acceptance as a complement to ERP therapy [52]. Given the nature of most compulsions associated with sexual-oriented OCD and the beliefs under sexual-orientation OCD, cognitive therapy can be particularly effective for patients with sexual obsessions [188], who tend to respond better than those with religious obsessions [52]. Cognitive behavioral therapy was reported to be a reliable alternative to SSRIs [189]. Increased pre-treatment limbic responsiveness has been related to better outcomes of CBT [141]. iCBT for OCD may be a viable option for individuals who experience treatment barriers [190]. EMDR was not found to be especially effective on OCD patients, in general, in comparison with other psychiatric conditions, although no study was found to use EMDR with sexual obsessions specifically [191]. However, the results suggest that there are positive effects of additive EDMR to exposure–response prevention therapy in patients with sexual obsessions, among others. In particular, in a sample investigating sexual-orientation related obsessions, a positive effect of association-splitting (a self-help technique derived from the semantic network approach) seems to be effective [84].

### 3.3. Compulsions Related to Sexuality in OCD Symptoms

#### 3.3.1. Compulsions Related to Sexual Obsessions

Traditionally, due to the scarce visibility of the compulsions predominant in OCD with sexual obsessions, the “pure obsessional type” of OCD, which is an OCD variant without compulsions, was discussed [22,192]. This concept has been questioned in recent decades, given the evidence of hidden compulsions in OCD with “repugnant”/morally based or unacceptable thoughts [22,49]. Sexual obsessions are often accompanied by mental rituals or compulsions [71,143] such as ruminations in order to seek an explanation for their intrusions or to ensure the patients is, for example, not a pedophile, mentally repeating words or images [22,92,114,142], demonstrating counting [92,134] and checking compulsions [66,105,106,134,163] (including somatic checking for signs of arousal) [23,66,193], seeking reassurance [22,194], and avoidance [193]. Sexual compulsions are also associated with hoarding [143,195,196], as well as with ordering and arranging compulsions [97], especially in patients with concomitant Tourette Syndrome, who, as stated before, usually have sexual obsessions [97].

Some studies suggested that compulsive sexual behavior is related to a need to relieve anxiety from sexual obsessions, initially met with resistance and control [5]; however, there is no evidence that permits establishing an association between compulsive sexual behavior and sexual obsessions [17,18,19,20,21].

#### 3.3.2. Compulsive/Addictive Sexual Behavior and OCD

“Compulsive sexual behavior”, also known as excessive sexual behavior [5], does not appear as a diagnostic entity in the DSM 5 but is recognized as an impulse control disorder in the ICD 11 [13]. This does not include compulsive behaviors, such as those belonging to the sphere of OCD-related disorders [5,49] It describes recurring sexually arousing fantasies and sexual urges that disrupt normal functioning, thus generating distress [5]. Online porn addiction, an arising diagnostic category that might have adverse effects in sexual development and sexual functioning, especially among the young population, could be included in this category [197].

It was divided into paraphilic and non-paraphilic sexual behavior, depending on the cultural acceptability of the behaviors involved [5]. Many behaviors have been included in the non-paraphilic category, from fixation with an unattainable partner to compulsive masturbation or compulsive use of the internet or erotica [198]. It often implies different psychiatric comorbidities such as mood disorders or substance use. Like many other compulsive behaviors, its relationship with OCD has been debated [199]. Some studies have found that patients with “problematic sexual behavior” share characteristics with both OCD [199] and addiction [200].

Paraphilic sexual behavior was related to OCD in the light of its response to SSRIs, as was shown by some case reports [14,15,16]. It was confronted by some studies that reported a mild response to medication compared to that of OCD patients [104,201]. Compulsive sexual behavior disorder and compulsive buying–shopping disorder have reported the highest scores on obsessive–compulsive general symptom scales according to one study [202] comparing OCD symptoms in diverse behavioral and impulsive disorders. Lifetime prevalences of compulsive sexual behavior disorder [199,203,204,205] and some statistically significant associations have been identified between both disorders [94]. Some authors suggest the existence of an obsessive–compulsive spectrum, which includes compulsive sexual disorder [199].

Some other authors have implied that such an association might only be based on a superficial resemblance between OCD and compulsive behaviors such as trichotillomania [5], noting the ego-dystonic nature of OCD obsessions in contrast with positive feelings that are frequently present in paraphilias [20]. Comparisons between guidelines and psychiatric criteria seem to give credit to the idea that, phenomenologically [206], and given its comorbidity with other impulsive disorders [199], compulsive sexual behavior could be compared to an addictive disorder [18,19,20,21,199,207], noting that reward deficiency syndrome, present in addictive disorders, is also associated with problematic sexual behavior [94] Patients with comorbid alcohol use disorder and OCD also present with a higher comorbidity of compulsive sexual behavior [208]. In one study, compulsive buyers were more likely to be diagnosed with sexual addiction [209]. Although higher rates of OCD have been found in samples with CBD than in controls, a meta-analysis on the co-occurrence of CBD with anxiety disorders found no significant correlation [210].

It seems to predominately affect men [199]. The heterogeneity of the group under the diagnostic label of compulsive sexual behavior has been signaled [199]. Compulsive sexual behavior has been correlated with lower white matter diffusivity in superior frontal brain regions compared to controls without the diagnosis [211].

Psychotherapy has been used in different modalities such as mindfulness-based relapse prevention therapy [212], aversion therapy, or desensitization; although it has been found to be effective, relapses are common [199]. Pharmacologic treatments include SSRIs and antiandrogens for dangerous cases, including medroxyprogesterone or cyproterone [199], or luteinizing hormone-releasing hormone agonists.

## 4. Discussion and Conclusions

This review highlights the significant impact of OCD on sexual functioning, with symptoms leading to decreased sexual satisfaction, dysfunction, and distress. The high prevalence of sexual dysfunction in OCD patients (39–77.5%) represents not merely a statistical finding but a profound human burden [30]. Beyond numerical estimates, these dysfunctions translate into distress, impairment in intimate relationships, and diminished quality of life. Recognizing this lived experience underscores the clinical importance of systematically assessing sexual functioning in OCD and addressing it as part of comprehensive treatment. Although acknowledging the limitations of the methodologically heterogenic samples and the variability in sample sizes, orgasmic dysfunctions seem to be the most prevalent among patients with OCD, significantly more prevalent than in other mental disorders related with anxiety [42] (See Table 1). Some authors, however, note that even though a prevalent and significant problem, sexual dysfunction, although perhaps taking different forms, is significantly prevalent in many other mental disorders with comparably significant prevalence [42,44]. In this review, no studies were found to be specifically designed to incorporate sexual dysfunction in sexual OCD as a separate entity. Sexual dysfunctions tend to be studied within OCD in general. Although contamination obsessions seem related to worse sexual functioning [24,34], no study, to our knowledge, has highlighted any specific relationship between sexual obsessions and sexual dysfunctions.

Surprisingly, a higher level of excitation in patients with OCD has been described, which contrasts with the general assumption of a generally decreased sexual functioning in OCD [32,33,34]. There is a contrast between this evidence and prevalent sexual dysfunctions detected by the more conventional scales (ASEX and others, see Table 2) [4,24,28,30,40,42,43,44,45,49]. It is important to note that the scales differ in their specific objects of study and conceptual frameworks: whereas the Dual Control Model addresses sexual excitement as a propensity towards sexual relationships, opposed by sexual inhibition -which is also increased in OCD-, while other scales measure the *functional* results of the balance between these two forces, in the form of sexual disfunctions. Furthermore, subtypes of sexual disfunction more closely associated with sexual excitement, including also arousal or desire impairments are not among the most prevalent in most of the samples (See Table 2). OCD patients seem to live in the tension between a higher sexual excitement and also strong factors of inhibition, which results in frequent sexual disfunctions.

The literature shows that sexual obsessions are among the most disturbing symptoms for patients with OCD [193], which lead to a high degree of shame [110], additional to the severity of the disorder itself. Although not directly measured as a mediating factor, it seems reasonable to assume that this very shame leads to the higher suicide rates found in this patient population [83,125,126]. The status of sexual OCD as a separate entity seems debated. On one hand, it is often associated with a younger age of onset and male gender, but the studies pointing at a distinct neurobiological basis, although significant, are still few. The same factors (gender, age) could at the same time point at social-psychological causes for symptom presentations in OCD. Some authors have also proposed that OCD symptoms may manifest from the typical worries which are most frequent in the age and gender group of the patients at their age of onset.

Although the subject has been debated during the last years, compulsive sexual behaviour seems to be, if not in name, conceptually separated from OCD, being mostly considered more similar to an addiction than a disorder of the OCD spectrum. We found no indication that OCD might be more associated with compulsions of a sexual content [22,52,196]. It is important to note that sexual OCD has a tendency to manifest mental or hidden compulsions (rather than being purely obsessional) which has also been implied as a cause for refractoriness to behavioral therapies by some authors.

Sexual obsessions are often linked to poorer outcomes, even though the results are not unanimous about refractoriness to some standard interventions, like the treatment with antidepressants [93,104,141,150,180,181,182]. Further statistical analyses of the available results could be valuable to support the association to treatment refractoriness to both cognitive/cognitive behavioral psychotherapy and pharmacological treatments. Emerging evidence of favorable responses to deep brain stimulation in patients with sexual obsessions illustrates that even the most severe presentations retain the potential for improvement [181]. Acknowledging both the challenges and the possibilities emphasizes the importance of incorporating sexual symptoms into treatment assessment and highlights avenues for more effective, individualized care. The high prevalence of sexual obsessions and compulsions, their impact, and the difficulties faced by many of the available treatment options such as cognitive behavioral therapy and selective serotonin reuptake inhibitors (SSRIs) [32,52,103] in these specific cases emphasize the necessity for specialized treatment approaches. However, sexual obsessions often predict treatment resistance, requiring specific therapeutic interventions [52,103,188].

The strengths of this review include its comprehensive scope, analyzing various dimensions of sexual function and symptoms in OCD patients. Our approach to the search, screening and data collection incorporates systematic elements, which we think provides methodological robustness. The studies assessed evidence from OCD patients of all ages and clinical presentations, thereby reflecting the heterogeneity of the disorder and its associated sexual manifestations. The inclusion of neurobiological and cognitive perspectives strengthens the understanding of these issues [88,148]. We have given, to the best of our knowledge, a vision of controversies related to sexual obsessions and compulsions, including the nature of the compulsions related to sexual obsessions and the categorization of compulsive sexual behaviors as areas of further debate and research.

Nevertheless, limitations exist. Despite incorporating certain elements of PRISMA guidelines, our work does not fulfill the requirements of a systematic review. Screening was performed by a single reviewer, and no independent assessment of study quality, risk of bias, or statistical heterogeneity was undertaken. Furthermore, the review aimed to provide a comprehensive overview rather than a quantitative synthesis of the evidence. These constraints should be considered when interpreting our findings, which are intended as a narrative integration of the available literature. The reliance on self-reported data [4] results in potential biases [4,30]. The heterogeneity in study methodologies (including different measuring tools and scales for assessing sexual disorders and OCD symptoms) makes it difficult to generalize the findings across populations [10,44]. Furthermore, the cultural and gender-specific factors influencing sexual symptoms in OCD require deeper investigation [67,109,194].

Future research should explore more precise diagnostic tools and targeted interventions to enhance the treatment efficacy and quality of life of patients affected by sexual symptoms in OCD. Many studies described sexual obsessions as part of a miscellaneous category including other kinds of taboo thoughts. Future research should consider sexual symptoms separately in the context of OCD. To our understanding, potential areas of interest for research include:

- The connections between sexual obsessions and impairment of sexual functioning in people with OCD.
- Specific social and gender-related factors behind sexual obsessions in OCD could better determine its etiology and perhaps bring a better understanding of these symptoms.
- Causes behind treatment refractoriness in sexual obsessions to gain more clarity beyond the conflicting results especially regarding cognitive therapies and treatment with SRIS.
- Gaining deeper insight into the efficacy of treatments like DBS against more traditional approaches for specific subgroups of OCD like sexual OCD could support the enhancement of clinical guidelines to provide a more individualized treatment.
- Specific psychotherapeutic approaches (Socratic dialogue, role-playing, acceptance and engaging therapy…) would benefit from more research in these specific cases, and possible provide evidence for structured therapeutical alternatives.

In conclusion, recognizing and addressing the sexual implications of OCD is crucial for comprehensive patient care. Effective interventions must incorporate both biological and psychological treatment strategies to improve sexual health and overall wellbeing in individuals with OCD.

## Figures and Tables

**Table 1:** Associations between different variables and the factor of the Dual Control Model: Sexual Inhibition due to fear of consequences or due to threat of performance failure, and Sexual Excitation: OCD: Obsessive–Compulsive disorder; Instruments: ASQ: -NA: Need for Approval, -DC: Discomfort with closeness, -C: confidence, -P Preoccupations with Relationships -R: Relationships as secondary; DPSS-R DS Disgust Propensity and Sensitivity Scale–Revised, evaluates disgust propensity (DP) and disgust sensitivity (DS); OBQ-46 Obsessive Beliefs Questionnaire46, -CT: control of thoughts, -IT importance of thoughts, -P: perfectionism, -RH: responsibility for harm, -RO: responsibility for omission; OCI-R: Obsessive Compulsive Inventory—Revised, -C: Contamination -T: Total; SIS: Sexual Inhibition Score: 1: due to threat of performance failure; 2: due to threat of performance consequences; SES: Sexual Excitation Score: Other acronyms: CI95: 95% Confidence Interval; GLM: Generalized Linear Model; M: Mean; r: Pearson’s correlation; SD: Standard Deviation; t: Student’s t; *: *p* < 0.05.

Gender	Measure: Value	Association (Measured Value)	Study
Associations with the Sexual Inhibition due to threat of performance failure (SIS1) subscale			
Both	OBQ-46: Perfectionism	β^ANCOVA^ = −0.013, t = −2.41 *	Pozza (2021) [33]
	DPSS-R: DS X contamination/washing symptoms	β^GLM^ (CI95 −0.069 −0.118 −0.021) χ^2^: 7.807 df = 1 *	Pozza, (2020) [36]
	SIS 2	r: 0.47 *	Pozza, (2019) [34]
	DPSS-r-DP	r: 0.29 *	Pozza, (2019) [34]
	DPSS-r-DS	r: 0.36 *	Pozza, (2019) [34]
	OBQ 46-P	r: 0.41 *	Pozza, (2019) [34]
	OBQ 46-RH	r: 0.31 *	Pozza, (2019) [34]
	OBQ 46-CT	r: 0.30 *	Pozza, (2019) [34]
	OBQ 46-RO	r: 0.30 *	Pozza, (2019) [34]
	OBQ 46-IT	r: 0.40 *	Pozza, (2019) [34]
Associations with the Sexual Inhibition due to fear of consequences (SIS2) subscale			
Women	ASQ-C	β^GLM^ = −0.070 * (Gender, OCI-R-T, ASQ)	Dettore, (2021) [37]
	ASQ-P	β^GLM^ = −0.108 * (Gender, OCI-R-T, ASQ)	Dettore, (2021) [37]
	Gender	β^GLM^ = −3.527 * (Gender, OCI-R-C, ASQ)	Dettore, (2021) [37]
	Gender and ASQ-R	β^GLM^ = 0.059 *, (Gender, OCI-R-C, ASQ)	Dettore, (2021) [37]
	Gender and OCIR-R-C	β^GLM^ = 0.963 *, (Gender, OCI-R-C, ASQ)	Dettore, (2021) [37]
Both	OCI-R-Checking	β = −0.10, t = −2.21 *	Pozza, (2019) [32]
	OCIR-CHECKING	β = −0.10, t = −2.21 *	Pozza, (2019) [32]
	DPSS-R: DS and contamination/washing symptoms	β^GLM^ (CI95): −0.070 (−0.120 −0.020) χ^2^: 7.626 df = 1 *	Pozza, (2020) [36]
	DPSS-R-DP	r: 0.28 *	Pozza, (2019) [34]
	DPSS-DS	r: 0.24 *	Pozza, (2019) [34]
	OCIR Checking	r: 0.24 *	Pozza, (2019) [34]
	OCIR Washing	r: 0.29 *	Pozza, (2019) [34]
	ASQ-NA	β^GLM^ = 0.036 * (Gender, OCI-R-C, ASQ)	Dettore, (2021) [37]
	ASQ-C	β^GLM^ = 0.044 *, (Gender, OCI-R-C, ASQ)	Dettore, (2021) [37]
	OCIR-R-C	β^GLM^ = 0.520 *, (Gender, OCI-R-C, ASQ)	Dettore, (2021) [37]
	ASQ-C	β^GLM^ = 0.046 * (Gender, OCI-R-T, ASQ)	Dettore, (2021) [37]
	ASQ-NA	β^GLM^ = 0.051 * (Gender, OCI-R-T, ASQ)	Dettore, (2021) [37]
Associations with the Sexual Excitement (SES) subscale			
Women	ASQ-DC	β^GLM^ = 0.051 * (Gender, OCI-R-T, ASQ)	Dettore, (2021) [37]
	Gender	β^GLM^ = −3.336 * (Gender, OCI-R-C, ASQ)	Dettore, (2021) [37]
	Gender and ASQ-C	β^GLM^ = −0.057 * (Gender, OCI-R-C, ASQ)	Dettore, (2021) [37]
	Gender and ASQ-DC	β^GLM^ = −0. 051 * (Gender, OCI-R-C, ASQ)	Dettore, (2021) [37]
Both	OCIR Washing	β = 0.12, t = 2.92 *	Pozza, (2019) [32]
	OCI-R: Total	β^ANCOVA^ = 0.014, t = 3.03 *	Pozza, (2021) [33]
	OCIR total	β^GLM^ = 0.012 * (Gender, OCI-R-T, ASQ)	Dettore, (2021) [37]
	Gender	β^GLM^ = −3.240 * (Gender, OCI-R-T, ASQ)	Dettore, (2021) [37]
	ASQ-DC	β^GLM^ = −0.030 * (Gender, OCI-R-T, ASQ)	Dettore, (2021) [37]
	ASQ-C	β^GLM^ = −0.030 * (Gender, OCI-R-T, ASQ)	Dettore, (2021) [37]
	ASQ-DC	β^GLM^ = −0.032 * (Gender, OCI-R-C, ASQ)	Dettore, (2021) [37]
	OCIR-R-T	r: 0.24 *	Mukhopadhyay, (2010) [38]

**Table 2:** General characteristics of the studies reporting prevalence rates of sexual dysfunctions in OCD samples. ASEX: Arizona Sexual Experience Scale; CAC: Compulsive Activity Checklist; CSFQ: Changes in Sexual Functioning Questionnaire; FSFI: Female Sexual Function Index; GRSSI: Golombok–Rust Sexual Satisfaction Inventory; IIEF-30: International Index of Erectile Function; MOCI: Maudsley Obsessional–Compulsive Inventory; N/A: not available; OCI-R: Obsessive Compulsive Inventory–Revised; QSD: Questionnaire for Screening Sexual Dysfunctions; Y-BOCS: Yale–Brown Obsessive Compulsive Scale. (>HC/PD): significantly more prevalent than in healthy controls or patients with panic disorder (no explicit prevalence value reported).

Study	Sample			Instruments Used		Global Prevalence		
	Total	Male	Female	Ocd	Sd	Total	Male	Female
Staebler, (1993) [26]	118	54	64	DSM III	N/A			
Monteiro & Noshirvani, (1987) [41]	46	25	21	N/A	N/A	54	64	43
Freund & Steketee, (1989) [4]	44	19	25	MOCL, CAC, DSM III	0–9 points Likert-Scale	39	25	
Van Minnen, (2000) [3]	14	-	14	N/A	QSD	76.4	-	76.4
Vulink, (2005) [43]	87	-	87	N/A	Scale based on ASEX and CSFQ	50	-	50
Kendurkar et al. (2008) [44]	50	28	22	DSM-IV	ASEX	50	28	22
Aksoy et al. (2012) [24]	40	N/A	N/A	N/A	GRSSI	-	-	24
Ghassemzadeh et al. (2016) [40]	56	20	36	OCI-R; MOCI	FSFI, IIEF-30	60.7	25	81
Zerdzinski et al. (2022) [30]	175	82	93	Y-BOCS	ASEX	66.6	54.2	77.5

**Table 3:** Prevalence rates of sexual dysfunctions involving infrequency, general satisfaction, aversion, avoidance, and desire in OCD samples. For more details about the samples and methods employed in each study, please see Table 1. (>HC/PD): significantly more prevalent than in healthy controls or patients with panic disorder (no explicit prevalence value reported).

Study	Infrequency			General Satisfaction			Aversion			Avoidance			Desire			Excitement		
	Total	Male	Female	Total	Male	Female	Total	Male	Female	Total	Male	Female	Total	Male	Female	Total	Male	Female
Staebler, (1993) [26]				63														
Monteiro & Noshirvani, (1987) [41]	3	8	4										16	13	9,5			
Freund & Steketee, (1989) [4]	25			73														
Van Minnen, (2000) [3]																		
Vulink, (2005) [43]						28									62			29
Kendurkar et al. (2008) [44]													13	28.6	22.8	24	25	22.8
Aksoy et al. (2012) [24]		57.1	63.6						24			60	-	-	60			
Ghassemzadeh et al. (2016) [40]				44.6	50	42							53	10	50			58
Zerdzinski et al. (2022) [30]																		

**Table 4:** Prevalence rates of sexual dysfunctions involving arousal, penile erection/vaginal lubrication, premature ejaculation, orgasm and orgasmic satisfaction and pain during intercourse in OCD samples. For more details about the samples and methods employed in each study, please see Table 1. (>HC/PD): significantly more prevalent than in healthy controls or patients with panic disorder (no explicit prevalence value reported).

Study	Arousal			Penile Erection/ Vaginal Lubrication			Premature Ejaculation			Orgasm			Orgasm Satisfaction			Pain		
	Total	Male	Female	Total	Male	Female	Total	Male	Female	Total	Male	Female	Total	Male	Female	Total	Male	Female
Staebler, (1993) [26]																		
Monteiro & Noshirvani, (1987) [41]					6			12		6	4	9						
Freund & Steketee, (1989) [4]				2				9		9						2		
Van Minnen, (2000) [3]																		
Vulink, (2005) [43]			25			25						33			20			
Kendurkar et al. (2008) [44]				26	21.4	31.8				46	46.4	45.4	28	35.7	18.2			
Aksoy et al. (2012) [24]			70					(>HC)							(>HC)			(>PD)
Ghassemzadeh et al. (2016) [40]				30.3	20	36				37.5	25	44			40			53
Zerdzinski et al. (2022) [30]				39	36.8	41.9				51	52.6	51.6			54.8			

**Table 5:** Summary of studies reporting sexual obsessions (SO) in patients with obsessive–compulsive disorder (OCD), including sample size, gender distribution (% women), clinical setting, and specific observations. Many studies do not separate sexual obsessions from symptom clusters which are described under “observation”. Prevalence by gender is presented when available. Abbreviations: SO = sexual obsessions; OCD = obsessive–compulsive disorder.

Authors	N	% Women	SO%	% Females	% Males	Setting	Observations
Aksaray, (2001) [45]	46	100	4	4	-	Outpatients	All-female sample
Alemany-Navarro, (2020) [77]	399	52	255	-	-	Outpatients	
Alonso, (2001) [78]	40	50	20	-	-	Outpatients	
Asadi, (2016) [79]	236	61	16.20	-	-	Outpatients	
Ay, (2018) [80]	67	52	16	-	-	Inpatients	3% without childhood trauma 30% with childhood trauma
Besiroglu, (2007) [81]	109	59	14.3	-	-	Outpatients	-
Çelebi, (2020) [82]	95	62.10	34.20	-	-	Outpatients	-
Cervin, (2022) [83]	500	55.80	1.90	-	-	Outpatients	“taboo obsessions”
Cherian, (2014) [70]	545	39	50.10	14.10	36.10	Outpatients	
Ching, (2017) [84]	120	68	39.66	-	-	Outpatients	College students Sexual orientation
Ching, (2021) [54]	592	69	18.80	-	-	Outpatients	-
Cifter, (2022) [85]	102	57.80	59.80	-	-	Outpatients	Children Harm/sexual
Cordeiro, (2015) [7]	75	16	5.01	-	-	Inpatients	-
Costa, (2012) [86]	901	-	55.40	-	-	Outpatients	-
Denys, (2004) [87]	335	62	5.90	-	-	Outpatients	Aggressive, sexual, and religious
Doron, (2014) [48]	157	45	12.50	-	-	Outpatients	Partner and sexual symptoms in OCD
Faragian, (2009) [88]	83	24.50	22	-	-	Inpatients	
Farrell, (2020) [89]	40	50	10	-	-	Outpatients	-
Ferrão, (2023) [90]	49	58	38.1	-	-	Outpatients	-
Freund, (1989) [4]	44	55	36		-	Outpatients	-
Grover, (2015) [91]	220	54	31	-	-	Inpatients	-
Grover, (2017) [92]	181	46.40	19.54	-	-	Outpatients	Patients with schizophrenia; aggressive, sexual, and religious obsessions; and counting items
Hasanpour, (2018) [93]	151	68	23	-	-	Outpatients	-
Hasler, (2005) [9]	317	58.20	76			outpatients	
Jiang, (2023) [94]	120	232	33.30	-	-	Outpatients	-
Kenezloi, (2018) [76]	102	30.40	7.70	-	-	Outpatients	-
Kim, (2014) [95]	57	33	33	-	-	Outpatients	-
Kuty-Pachecka, (2021) [52]	313	-	16.80	50	65	Outpatients	Meta-analysis
Labad, (2008) [64]	193	38	22,6	5.60	23.70	Outpatients	Sexual/religious
Lochner, (2009) [96]	606	52.30	70	-	-	Outpatients	-
Mack, (2010) [97]	318	50	18.30	-	-	Outpatients	-
Maina G, (2007) [98]	204	50	22.10	-	-	Outpatients	-
Mataix- Cols, (2008) [71]	238	36.80	28	18.40	33.60		
Mathis, (2011) [65]	215	47	88				Cluster including aggressive, sexual, religious, somatic obsessions, and checking compulsions
Monzani, (2015) [55]	189	45.50	63	-	-	Outpatients	Forbidden thoughts
Rosa, (2012) [99]	815	58.30	4.18	-	-	Outpatients	-
Selles, (2014) [100]	292		7.1 16.60	-	-	Outpatients	Younger youth Older youth
Shahzad, (2020) [101]	200	55.50	50.50	-	-	Inpatients	-
Sharma, (2014) [102]	802	38	28.80	-	-	Outpatients	-
Siev, (2011) [103]	15	53	39	-	-	Outpatients	-
Stein, (2007) [104]	434	54	18.70	-	-	Outpatients	-
Stewart, (2007) [105]	231	67	16.10	-	-	Outpatients	
Stewart, (2008) [106]	83	24.50	22	-	-	Outpatients	-
Storch, (2008) [107]	74	29	6.06	-	-	Outpatients	Children
Torres, (2011) [108]	582	56.4	51.40	-	-	Outpatients	Sexual/religious
Torres, (2016) [109]	1001	56.80	57.10	-	-	Outpatients	Sexual/religious
Torresan (2013) [10]	858	58.70	55.70	52.20	60.70	Outpatients	
Torresan et al. (2009) [69]	330	45	33.60	27.39	39.27	Outpatients	-
Visvalingam, (2022) [110]	53	74.50	7.27	-	-	Outpatients	-
Viswanath, (2011) [75]	84 80	-	14.3 30.0	-	-	Outpatients	Familial OCD Sporadic OCD
	164	-	14	-	-		Total
Wang, (2012) [111]	275 327	33.10 52.60	29.80 18	-	-	Outpatients	Early-onset OCD Late-onset OCD
Williams, (2013) [112]	83	56.80	12.20	-	-	Outpatients	-
Wu, (2018) [113]	215	43	92 76	-	-	Outpatients	Symptoms of severity No symptoms of severity “aggressive, sexual, somatic, and checking”

**Table 6:** Other obsessions and compulsions associated with sexual obsessions.

Author	Year	Number of OCD Patients (n)	Mean Age	Statistical Analysis	Total Variance Explained by the Model %	Total Number of Factors	Factor Number for Sexual Obsessions	Variance Explained by Factor	Associated Obsessive Symptoms	Associated Behavioral (Compulsive) Symptoms
Baer [144]	1994	107	Adults	Varimax	57	3	3	11.3	Aggression, religious	-
Hantouche [150]	2003	615	Adults	Varimax		3	2		Aggressive, religious, miscellaneous	Miscellaneous
Leckman [151]	1997	292	Adults	Varimax	63	4	1	30.1	Aggressive, religious, somatic	Checking
Mataix-Cols [152]	1999	354	Adults	Varimax	65	5	5	9.7	Religious	-
Tek [153]	2016	45	Adults	Varimax	56	5	4	9.7	Religious	-
Girishchandra [154]	2001	202	Adults	Varimax	35	5	5	4.2	Religious	-
Mataix-Cols [155]	2002	153	Adults	Varimax	64	5	5	7.9	Somatic	
Cavallini [156]	2002	180	Adults	Varimax	60	5	3	11.5	Aggressive, somatic, religious	Checking, repeating
Bezahler [27]	2024	160	Adults	Varimax	44	4	4	11.8	Religious	-
Denys [87]	2004	335	Adults	Varimax	41	5	2	9.8	Aggressive, religious	
Denys, de Geus [66]	2004	150	Adults	Varimax	42.5	5	1	14.5	Aggressive, religious	
Hasler [9]	2005	169	Adults	Varimax	63	4	1	19.5	Aggressive, religious, somatic	Checking
Kim [95]	2005	124	Adults	Varimax	62	4	3	10	Aggressive	
Delorme [157]	2006	73	Child	Varimax	78	4	2	13	Aggressive, somatic	Counting
Mckay [158]	2006	137	Child	Oblimin	68	4	3	12.7	Contamination, aggressive, magical thoughts, somatic, religious, symmetry	Repeating, counting, rituals involving others
Pinto [146]	2007	293	Adult	Varimax	76.4	5	5	7.4	Aggressive, religious	
Cullen [159]	2007	221	Adult	Varimax	-	4	1	-	Aggressive, religious, somatic	
Hasler [9]	2005	418	Adult	Promax	63.7	4	1	17.7	Aggressive, religious, somatic	Checking
Stein [104]	2007	434	Adult	Varimax	-	5	3	-	Aggressive, religious	
Stewart [105]	2007	231	Child	Promax	66.6	4	4	9.1	Religious	
Mataix-Cols [71]	2008	238	Child	Varimax	54.8	4	2	13.7	Aggressive, religious	
Bloch [147]	2008	5124	General	Varimax, meta-analysis	79	4	2	21	Aggressive, religious, somatic	
		4445	Adult							
		679	Child		81.7	4	4	16.4		
Faragian [88]	2009	110	Adult	Varimax	58.7	5	1	15.9	Religious, aggressive	Counting
Højgaard [160]	2017	854	Child	Varimax	-	3	1	-	Aggressive	Checking

## Abbreviations

ASQ	DPSS-R DS Disgust Propensity and Sensitivity Scale–Revised, which evaluates disgust propensity (DP) and disgust sensitivity (DS).
GAD	Generalized Anxiety Disorder.
GLM	Generalized Linear Model.
OBQ-46	Obsessive Beliefs Questionnaire 46.
OCI-R	Obsessive Compulsive Inventory—Revised.
PD	Panic Disorder.
SO	Sexual Obsessions.
SD	Sexual Dysfunction.
SES	Sexual Excitation Score.
SIS	Sexual Inhibition Score—1: due to threat of performance failure; 2: due to threat of performance consequences.
r	Pearson’s Correlation.
SD	Standard Deviation.
SGRS	Sexual Guilt Rating Scale.
t	Student’s t.
